# Barriers to Uptake of Open-Source Automated Insulin Delivery Systems: Analysis of Socioeconomic Factors and Perceived Challenges of Caregivers of Children and Adolescents With Type 1 Diabetes From the OPEN Survey

**DOI:** 10.3389/fcdhc.2022.876511

**Published:** 2022-07-25

**Authors:** Antonia Huhndt, Yanbing Chen, Shane O’Donnell, Drew Cooper, Hanne Ballhausen, Katarzyna A. Gajewska, Timothée Froment, Mandy Wäldchen, Dana M. Lewis, Klemens Raile, Timothy C. Skinner, Katarina Braune

**Affiliations:** ^1^ Department of Paediatric Endocrinology and Diabetes, Charité—Universitätsmedizin Berlin, Berlin, Germany; ^2^ School of Public Health, Physiotherapy & Sports Science, University College Dublin, Belfield, Ireland; ^3^ School of Sociology, University College Dublin, Belfield, Ireland; ^4^ Berlin Institute of Health (BIH), Berlin, Germany; ^5^ #dedoc° Diabetes Online Community, Dedoc Labs GmbH, Berlin, Germany; ^6^ Population Health Sciences, Royal College of Surgeons in Ireland, Dublin, Ireland; ^7^ OpenAPS, Seattle, WA, United States; ^8^ Department of Psychology, University of Copenhagen, Copenhagen, Denmark; ^9^ Australian Centre for Behavioural Research in Diabetes, Melbourne, Australia; ^10^ La Trobe University, Bendigo, Australia; ^11^ Institute of Medical Informatics, Charité—Universitätsmedizin Berlin, Berlin, Germany

**Keywords:** automated insulin delivery, closed-loop, digital innovation, diabetes technology, barriers, human factors, health inequalities, access

## Abstract

**Background:**

As a treatment option for people living with diabetes, automated insulin delivery (AID) systems are becoming increasingly popular. The #WeAreNotWaiting community plays a crucial role in the provision and distribution of open-source AID technology. However, while a large percentage of children were early adopters of open-source AID, there are regional differences in adoption, which has prompted an investigation into the barriers perceived by caregivers of children with diabetes to creating open-source systems.

**Methods:**

This is a retrospective, cross-sectional and multinational study conducted with caregivers of children and adolescents with diabetes, distributed across the online #WeAreNotWaiting online peer-support groups. Participants—specifically caregivers of children not using AID—responded to a web-based questionnaire concerning their perceived barriers to building and maintaining an open-source AID system.

**Results:**

56 caregivers of children with diabetes, who were not using open-source AID at the time of data collection responded to the questionnaire. Respondents indicated that their major perceived barriers to building an open-source AID system were their limited technical skills (50%), a lack of support by medical professionals (39%), and therefore the concern with not being able to maintain an AID system (43%). However, barriers relating to confidence in open-source technologies/unapproved products and fear of digital technology taking control of diabetes were not perceived as significant enough to prevent non-users from initiating the use of an open-source AID system.

**Conclusions:**

The results of this study elucidate some of the perceived barriers to uptake of open-source AID experienced by caregivers of children with diabetes. Reducing these barriers may improve the uptake of open-source AID technology for children and adolescents with diabetes. With the continuous development and wider dissemination of educational resources and guidance—for both aspiring users and their healthcare professionals—the adoption of open-source AID systems could be improved.

## Introduction

There are about 1.2 million children and adolescents <20 years of age worldwide who live with type 1 diabetes (1). To reduce their risks of acute and long-term complications, therapeutic guidelines recommend target hemoglobin A1c levels of <7,0% (2, 3). However, not all children and adolescents achieve these glycemic targets. Methods for treating diabetes vary widely (4, 5). Technology is evolving rapidly and continuously, which is significant in improving health conditions. Medical devices, mobile technology, cloud computing, and social media make it possible—especially for patients—to improve, co-design, and co-develop new treatments (6). This possibility is particularly important for children and adolescents living with diabetes, as well as their caregivers and families (7).

Automated Insulin Delivery (AID) systems represent an important advance in diabetes therapy. Given the limitations in access to this technology, the #WeAreNotWaiting community has created so-called “Do-It-Yourself Artificial Pancreas Systems” (DIYAPS) or ‘open-source AID’ systems and made the resources needed to build them available *via* open-source platforms (7–10). These systems are not approved by regulatory bodies and thus are used by people with diabetes at their own risk. However, devices that are approved and commercially available are needed as components (e.g. insulin pumps and continuous glucose monitoring (CGM) systems). There are several types of open-source AID systems each with multiple different branches. While OpenAPS runs the algorithm on a Linux-based minicomputer, AndroidAPS, Loop, and FreeAPS are smartphone applications. Depending on the setup, additional hardware (e.g. OpenAPS rig, Riley-, Orange- or EmaLink) and software components (e.g. xDrip+, Nightscout) may also be required (11, 12). The algorithms for automated insulin delivery adjust insulin dosing according to the user’s glycemic levels. Previous studies have shown that open-source AID systems have the potential to improve clinical outcomes in users of several age groups (e.g. better HbA1c-level and time in range (TIR)) (13–23). Moreover, they reduce the individual burden of living with diabetes, such as improving quality of life and sleep quality (14, 24–28). A profound understanding of pump therapy and CGM systems, but also technical literacy are needed to successfully build and use open-source AID (25, 29–32). The questions that arise include who is actually able to use and even create this technology and who would benefit from it (33, 34).

Few studies have examined the perceived barriers to adopting open-source AID solutions. Schipp et al. looked at the perceived challenges of adults during the set-up of their AID system (25). However, this work and most other research reports almost exclusively focus on the experiences and emotions of people with diabetes who have already successfully built and are using an open-source system. Among members of online support groups such as “Looped” on Facebook (N=28,323), there are a number of people with diabetes and caregivers of children who have not yet built and used open-source AID. O’Donnell et al. previously identified barriers perceived by adult non-users (35), but evidence is currently lacking concerning children and adolescents with diabetes and their caregivers. Therefore, it might be possible that the barriers to building and using open-source AID are not completely identified yet. To fill such an evidence gap, this paper refers to the caregivers of children living with diabetes. One of the challenges to addressing this gap is that to respond to questions about the barriers to using open-source AID systems, it is necessary to recruit caregivers who know about these systems and have some understanding of what it entails to build and maintain them. Therefore, this study aimed to recruit caregivers from the #WeAreNotWaiting community who meet these criteria. Clearly, with members of this well-informed and pro-active community, it is to be expected that there are biases with this sample. Hence, our results will not include the barrier of not knowing about the systems.

The overall aim of this study is to 1) investigate the barriers to scale-up open-source AID systems in caregivers of children and adolescents in the #WeAreNotWaiting community who are non-users of open-source AID and 2) analyze the participants’ socioeconomic status in relation to the perceived barriers.

## Methods

### Study Design

This survey was part of a large retrospective, multinational, web-based cross-sectional study conducted from September to November 2020 with users and non-users of open-source AID within the #WeAreNotWaiting community. Two questionnaires, titled “Your Thoughts about DIYAPS” (DIWHYnot) and “About you and your child” (socioeconomic factors) were distributed to caregivers of children with diabetes who were still non-users of open-source AID.

### Survey Tool

Questionnaires were designed by an interdisciplinary team of researchers living with type 1 diabetes and were both users and non-users of open-source AID (35); some researchers had used open-source AID for several years, some were in the process of uptaking systems, and others were not interested in using open-source AID. TF—a non-user—provided statements about challenges regarding the set-up, which were reviewed and completed by users (SO, DL, KB, MW) and non-users (KAG) to generate a final list of items. The ‘DIWHYnot’ questionnaire comprised of a combination of check-box items with comments, and questions on a 5-point Likert scale (“Strongly Agree”, “Agree”, “Neither Agree or Disagree’’, “Disagree” and “Strongly Disagree”); respondents were able to choose “Other”, “I don’t know” or “I’d rather not say” in response to the questions (Appendix A). The questionnaire applied branching logic to address progressively more specific questions. “About you and your child” used mostly check-box items and open-field inputs to collect information on socio-economic factors; respondents were again capable of answering “I’d rather not say”, “Other”, “None of the above’’ or “I don’t know”, allowing each participant to be included in the statistics (Appendix B).

### Participants and Recruitment

Caregivers (e.g. a parent, family member, or legal guardian) of children and adolescents under the age of 18 who are living with diabetes were eligible for participation. The participants were recruited *via* Facebook groups including the multinational “Looped” groups, “AndroidAPS users”, “CGM in the Cloud” and “Nightscout Germany”; through the OPEN website; and social media accounts such as “Diabetes Daily”. The survey was conducted using the REDCap electronic data capture tool hosted by Charité – Universitätsmedizin Berlin. Ethical approval for the survey—including all questionnaires—was granted by the Life Sciences Human Research Ethics Committee at University College Dublin (LS-20-37).

### Data Analysis

After data cleaning, analyses were conducted using IBM SPSS Statistics 27 (International Business Machines Corporation, Armonk, NY, United States) and Microsoft Office (Microsoft Corporation, Redmond, WA, United States). Validity and internal consistency (e.g. factor analysis, Cronbach’s alpha) tests of specific survey items were performed followed by descriptive and inferential analyses (e.g. Levene’s test, independent samples t-test).

## Results

### Participant Characteristics

Of the 1052 total participants of the OPEN study, 56 were caregivers of children with diabetes who were not using open-source AID at the time of data collection (**Supplementary Figure 1**). Responses from 49 participants were included in the analysis of socioeconomic factors (of children and caregivers). Overall, 59.2% of the children were male with a mean age of 11 years (range: 1-18 years, SD: 3 years). The participants were from 13 different countries of which 67.3% were from Germany (n=12), Denmark (n=11), the United Kingdom (n=5), and the United States (n=5). Of the participants, 87.8% described the ethnicity of the children as “White”. 63.3% of the caregivers were employed either full- or part-time, mostly in the science sector (32.6%), most commonly with educational qualifications of a Master’s (34.7%) or Bachelor’s degree (30.6%). The majority reported annual household income ranged from 100 000 to 199 999 US dollars (32.7%) and 50 000 to 99 999 US dollars (20.4%). **Supplementary Table 1** summarizes the demographic data in detail. Most participants (84.6%; n=22/26) expressed they wanted to learn more about open-source AID, especially regarding the support they would get if they decided to build a system. More than half of them (64.3%; n=18/28) have not yet created a system but could imagine doing so under certain conditions. Among those who could imagine building an open-source AID system, most (30.4%; n=17/56) were interested in “Loop” (app for Apple iPhones). While 61.5% (n=16/26) were convinced and wanted to create a system, a smaller group (21.4%; n=6/28) were already in the process of setting up a system but had not yet used it. 28.6% (n=16/56) did not report out-of-pocket expenses for the required diabetes equipment, while the remaining participants pay up to 50 USD per month (7.1%; n=4/56), rarely more. Expenses for insulin were reported most often (14.3%; n=8/56), followed by CGM sensors (10.7%; n=6/56). When asked how the participants had heard about open-source AID, the majority responded “I have heard of it through social media” (48.2%; n=27/56).

### Types of Barriers

Only a few respondents (11.1%; n=3/27) perceived the necessary components were too expensive. 33.3% (n=9/27) were interested in building an open-source AID but did not know where to source some of the components, especially additional components, such as the RileyLink and OpenAPS rig (77.8%; n=7/9) and loopable pumps (66.7%; n=6/9).

Overall, the result of the internal consistency analysis was acceptable (Cronbach’s alpha=0.741). 18 out of 20 items questioned all perceived barriers except for the procurement and the costs of necessary components to use open-source AID (Cronbach’s alpha=0.730). The reliability of the remaining 18 items was improved by deleting several statements, with Cronbach’s alpha increasing to 0.807. Following the elimination of the items “insufficient expertise of diabetes teams”, the “missing knowledge about pump therapy” and the “imagination to carry the required equipment”, a good internal consistency was achieved. The remaining 15 items were examined through exploratory factor analysis using principal component analysis and the Varimax rotation method, which indicated the point of inflection on the screen plot was at three factors and this generated a simple solution (factors only loading > 0.4 on one factor (**Table 1**). As the results suggested, the 15 items can be reduced to three components (cumulative proportion=60.71%): Dimension 1 “building and maintenance” of a system, Dimension 2 “therapy knowledge and trust in technology” and Dimension 3 “support and liability”. The item “My child is currently using commercial automated delivery systems.” (loading<0.4) did not fit into any of the dimensions. Thus, our final scale was best represented by the remaining 14 items in **Table 1**.

**Table 1:** Item Factor Loadings > 0.4 for Final 14 Items in the Barriers to Uptake Questionnaire.

Items	Dimension 1	Dimension 2	Dimension 3
I don’t have sufficient knowledge of CGM therapy.		0.619	
My child is currently using commercial automated delivery systems.			
I don’t trust machines/technologies to take over the control of my child’s diabetes.		0.761	
I don’t trust open-source technology.		0.709	
I don’t trust products that are not approved by a regulatory body.		0.737	
I don’t have the necessary programming knowledge to build the software on my own.	0.867		
I can find help to build the DIYAPS but I am scared I won’t be able to maintain it.	0.773		
I am afraid we might lose the support of my child’s healthcare provider if we start looping.			0.647
I am afraid we might lose my child’s health insurance if we start looping.			0.776
I feel it would increase my level of responsibility and I don’t want that.		0.522	
I feel it would increase my level of liability and I don’t want that.			0.503
I don’t have the energy to do it myself.	0.917		
I don’t have the time to do it myself.	0.833		
I feel that the DIYAPS expertise and resources are too overwhelming to understand.	0.871		
My child’s diabetes team discourages me from building a DIYAPS.			0.845

Extraction Method: Principal Component Analysis.

Rotation Method: Varimax with Kaiser Normalization.

**Figure 1:**
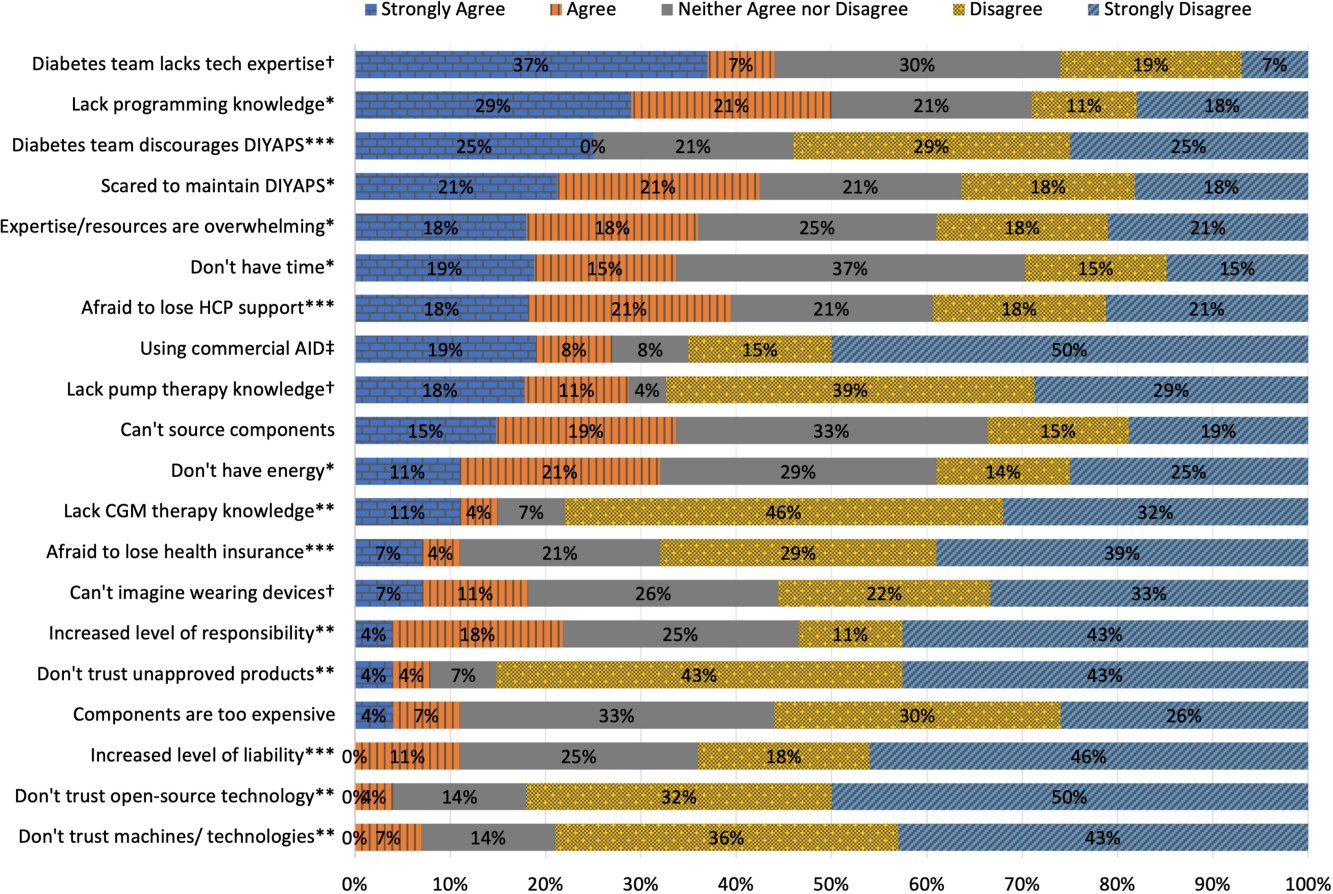
N=56; responses to statements regarding interest in building an open-source AID (“I would be interested in building a DIYAPS, but…”). Participants rated statements on a 5-point Likert scale (strongly agree, agree, neither agree nor disagree, disagree, strongly disagree). The responses were classified into three dimensions using principal component analysis and are labeled accordingly (*Building and Maintenance, **Knowledge and Trust in Technology, ***Support and Liability, † were excluded due to reduced reliability, ‡ was excluded after principal component analysis due to insufficient loading).

Regarding “building and maintenance” of the open-source AID system, half of the participants reported (n=14/28) that they did not perceive having the skills needed to build it. 42.9% (n=12/28) knew they could find help to set it up, but were not sure if they would be able to maintain the system. Lack of time (33.3%; n=9/27) and too little energy (32.1%; n=9/28) were concerns, in addition to that resources are too overwhelming to understand (35.7%; n=10/28). In terms of “therapy knowledge and trust in technology”, respondents were most likely to fear having to take on additional responsibility (21.4%; n=6/28). Insufficient knowledge about CGM was reported by 14.3% (n=4/28). Only a minority of the respondents (7.1%; n=2/28) reported a lack of trust in machines and technologies to take over the control of diabetes in general. Similarly, only one respondent reported a lack of trust in products that have not been approved by a regulatory body. As for “support and liability”, by far the biggest concern was a potential loss of support by the healthcare provider (39.3%; n=11/28), followed by discouraging the uptake of an open-source AID by the diabetes team (25%; n=7/28). There was less agreement on both the fear of losing health insurance if they start looping and the worry that liability would increase (10.7%; n=3/28). Additionally, it is important to mention that 44.4% (n=12/27) reported no available support from healthcare professionals (HCP) of the diabetes care team due to their limited expertise in diabetes technology in general (**Figure 1**).

### Encouragement

It is remarkable that many of the respondents would be encouraged to set up an AID system if their decision was officially supported. For example, 88.9% (n=24/27) confirmed “strongly agree” or “agree” that they would be motivated to start an open-source AID if their HCP recommended it. A similar number (80.8%; n=21/26) would be convinced if professional diabetes associations such as the International Diabetes Federation supported their use. Both the support of diabetes care teams (77.8%; n=21/27) and increased uptake in open-source AID in healthcare in general (74.1%; n=20/27) would encourage some caregivers to consider taking up a system. However, for slightly more than half of the participants (55.6%; n=15/27), it is also important that there is a company to provide warranty and support in case of technical errors.

### Differences by Education Level and Household Income

To determine whether perceptions of different barriers are related to educational attainment, participants with Bachelor’s and Master’s degrees, as well as different household income groups were compared (**Table 2**).

**Table 2:** Comparison of education and annual household income level of caregivers of children and adolescents who are non-users of open-source AID.

	Education			Annual income		
Item	Bachelor’s degree	Master’s degree	t-test	USD50 000 to 99 999	USD100 000 to 199 999	t-test
…I want to build a DIYAPS system.	7	10	0.398	8	10	0.358
…it is too expensive to purchase all the components.	8	10	0.654	8	10	0.833**
…I don’t know where to source all the components.	8	10	-0.105	8	10	-0.274
I don’t have sufficient knowledge of CGM therapy.	8	11	-0.589	8	10	0.147
I am currently using commercial automated delivery systems.	7	10	1.286**	8	9	1.039
I don’t trust machines/technologies to take over the control of my diabetes.	8	11	-0.408	8	10	-2.074
I don’t trust open-source technology.	8	11	-1.657	8	10	0.000
I don’t trust products that are not approved by a regulatory body.	8	11	-0.986	8	10	0.622
I don’t have the necessary programming knowledge to build the software on my own.	8	11	1.050	8	10	1.805
I can find help to build the DIYAPS but I am scared I won’t be able to maintain it.	8	11	0.691	8	10	2.855*
I am afraid I might lose the support of my healthcare provider if I start looping.	8	11	-1.361	8	10	1.155
I am afraid I might lose my health insurance if I start looping.	8	11	0.975	8	10	0.457
I feel it would increase my level of responsibility and I don’t want that.	8	11	-2.904*	8	10	0.805
I feel it would increase my level of liability and I don’t want that.	8	11	-1.095**	8	10	1.042
I don’t have the energy to do it myself.	8	11	-0.263	8	10	0.041
I don’t have the time to do it myself.	8	10	0.147	8	9	-0.178
I feel that the DIYAPS expertise and resources are too overwhelming to understand.	8	11	-0.392	8	10	-1.452
My diabetes team discourages me from building DIYAPS.	8	11	-1.288	8	10	0.989
How much do you have to pay for the diabetes supplies monthly?	8	11	1.966**	8	10	0.300

*t-test for Equality of Means, p<0.05.

**Levene-test for Equality of Variance, p<0.05.

The worry about having to take on additional responsibility when using open-source AID differed significantly between caregivers with a Bachelor’s and a Master’s degree (T-test, T=-2.904, df=17, p=0.01), with participants with a Master’s degree being more aware. No significant differences were found regarding the caregivers’ motivation to build open-source AID (T-test, T=0.398, df=15, p=0.69) or concerns about sourcing components (T-test, T=-0.105, df=16, p=0.92). Differences were also found between the income groups of USD 50 000 to 99 999 and USD 100 000 to 199 999 per year. The worry of not being able to regularly maintain the open-source AID system once they have successfully built it was perceived as more significant for caregivers with a higher income (T-test, T=2.855, df=16, p=0.01). Non-significant differences were found for insufficient programming skills (T-test, T=1.805, df=16, p=0.09) and fear of losing support from the healthcare provider (T-test, T=1.155, df=16, p=0.27).

## Discussion

Non-users of open-source AID reported several structural and individual barriers to the adoption of open-source AID. Structural barriers concerned the sourcing of compatible insulin pumps and additional components. Major individual barriers were limited perceived technical skills such as programming knowledge, limited support by medical professionals, and therefore the concerns of not being able to build and maintain the AID system by themselves. However, it was neither the confidence in the open-source technologies and lack of their regulatory approval nor the fear of digital technologies taking over the control of diabetes management that prevented non-users from creating and using an open-source AID system. Except for the two significant characteristics of annual household income and highest educational degree, socioeconomic status did not have a significant impact on the perceived barriers.

The structural problem of obtaining suitable insulin pumps could best be explained by the fact that not all compatible models are available *via* prescription. A compatible insulin pump refers to one that can interoperate with the algorithm and receive commands to adjust insulin delivery. Although the number of compatible insulin pump models has increased in recent years, some “loopable” pumps are only available on prescription in select countries, insurances often set time limits when a prescription can be renewed, and the availability of older out-of-warranty pump models, e.g. traded second-hand *via* online platforms, is limited. A previous study on barriers to the adoption of insulin pumps in Ireland has identified some people with diabetes having difficulties in orienting and understanding the health systems and their reimbursement principles (36). In terms of individual challenges, the self-perceived insufficient programming skills emerged as very relevant.

Finally, non-users were concerned about their ability to maintain and service the system on their own. Previous studies describing the experiences of those who have successfully set up open-source AID have shown that peer-support can help overcome this barrier (25, 37) with the support of experienced or technically versed community members. Their fears and worries could be alleviated and their self-confidence and determination could be strengthened through the achievement of successfully setting up an AID system. Therefore, connecting with other members of the #WeAreNotWaiting community online or in-person, as well as utilizing the available resources (e.g., online documentation and tutorials) could help close pre-existing knowledge gaps.

Similarly, respondents reported not receiving the desired support from their diabetes care teams, e.g., as they have limited necessary expertise in diabetes technology in general and open-source AID in particular. Medical professionals are important gatekeepers when it comes to access to open-source AID and, in fact, all diabetes technology. Many families with children with diabetes experience difficulties in access to AID systems, i.e., because these are not yet approved in their countries, are not approved for children of a particular age or are not reimbursed. It may take many years until AID technology is fully affordable and accessible for everyone. Until such time, open-source AID will continue to “fill the gap” for some people with diabetes in accessing this life-enhancing technology and therefore merits the support of medical professionals as well as other stakeholders in the diabetes community. Previous work has highlighted that HCPs are caught in a dilemma between the uncertainty regarding liability, the lack of regulatory approval of open-source AID, and supporting the choice and best interest of their patients (12, 33, 38). Meanwhile, individuals with diabetes may also face an associated dilemma between the advantages of using an AID system and the risk of losing support from HCPs. It seems that the open-source innovations have gained more acceptance among some HCPs, but not the majority of them. Due to the trend, more knowledge about the technology is available and can be used for supporting patients who are interested in applying an AID system (39). Therefore, changing medical guidelines to support open-source AID could help to reduce concerns of people with diabetes and remove the two barriers “insufficient expertise of diabetes teams” and the associated “lack of support”. This point of view has already been supported by other authors who looked at the perspectives of HCPs (34, 39–42). A recently published international consensus statement provided practical guidance to HCPs and specifically addressed professional educational aspects but also ethical and legal issues (12). For children and adolescents specifically, the consensus group recommended that the child’s welfare should always be considered by HCPs and caregivers who are setting up open-source AID for children, with the child’s assent and engagement (12). Further research should investigate the experiences and thoughts of HCPs and particularly address the challenges of procuring necessary devices (insulin pumps, CGM) *via* prescription (12, 39, 43–47).

To the best of the authors’ knowledge, this is the first study that is determining the perceived challenges in detail that are seen by caregivers of children and adolescents with diabetes when setting up an open-source AID. Previous studies have specifically examined the experiences of adult users (less so children) with open-source AID (14, 17, 25, 37, 48, 49). The fact that most of the involved researchers have personal experience with open-source AID as active users in addition to their professional roles, as well as the involvement of non-users in the study design underlines the public and patient engagement as a strength of this paper. Of further strength is the multinational character of this study. Nevertheless, several limitations apply as the sample size of 56 participants is relatively small compared to other sub-cohorts that participated in the OPEN survey. Furthermore, the participants identified predominantly as “White”, based in North America and Europe, and mostly had a high socioeconomic status. The number of participants with an educational level lower than a Bachelor’s degree was too small to be included in the factor analysis. Therefore, the sample is not representative of all caregivers of children with diabetes who are not using an open-source AID system, and the results may not reflect the non-user population of developing and emerging countries (50). Finally, based on the results of the reliability and validity tests, the items of the questionnaires could be adapted and improved for further surveys. Given the smaller sample size and socioeconomic background of this specific population, it would be of interest to investigate barriers in non-users outside the #WeAreNotWaiting community. Previous studies described motivations, enablers, and sources of support in open-source AID users (adults and caregivers of children) (14, 25, 27). While this paper describes barriers that prevent non-users from building open-source AID, it may be equally important to investigate what might encourage them to do so.

Lastly, as commercially developed AID systems have recently become available in select countries and can be made available *via* prescription, it would be of interest to investigate barriers to uptake with respect to commercial AID systems and outside the context of the #WeAreNotWaiting community as well.

As part of a retrospective, multinational, web-based cross-sectional study of the #WeAreNotWaiting community, this work has identified caregivers’ challenges for uptaking an open-source AID system. In order to increase the distribution of open-source AID, using online resources and community peer-support could be useful and complement support from medical professionals. Sharing problems that occurred during the build and use of open-source AID, and how these were encountered, could be insightful to aspiring users. The current open-source AID documentation already includes a comprehensive list of build errors and solution strategies. The findings of this study could help the #WeAreNotWaiting community to further extend these resources to better meet the needs of current non-users. In addition, providing educational resources to HCPs, such as the recently published consensus statement, could also help care teams to understand and better support current and future open-source AID users. Finally, regional differences and limitations in the availability of insulin pumps and CGM systems as AID components should be addressed by manufacturers, regulators, and policymakers. If access to diabetes technology would be more equal, many more people with diabetes would be able to benefit from digital innovations.

## Data Availability Statement

The raw data supporting the conclusions of this article will be made available by the authors, without undue reservation.

## Ethics Statement

The studies involving human participants were reviewed and approved by Life Sciences Human Research Ethics Committee at University College Dublin. The patients/participants provided their written informed consent to participate in this study.

## Author Contributions

TF, KG, SO’D, DL, MW and KB created the survey design. Ethics approval was sought by SO’D. AH, YC, DC, SO’D, HB and KB processed and analyzed the data. AH and KB wrote the initial draft of the manuscript. All co-authors had access to the full data set, and have critically reviewed and revised the manuscript, approved the final version of the manuscript and can confirm the integrity of the study.

## Funding

The authors declare that this study received funding from European Commission's Horizon 2020 Research and Innovation Program under the Marie-Sklodowska-Curie Action Research and Innovation Staff Exchange (RISE) grant agreement number 823902, the DFG-funded Digital Clinician Scientist Program of the Berlin Institute of Health (BIH), and the SPOKES Wellcome Trust Translational Partnership Program. The funders was not involved in the study design, collection, analysis, interpretation of data, the writing of this article or the decision to submit it for publication.

## Conflict of Interest

Authors HB, KG and TF were employed by company Dedoc Labs GmbH.

The remaining authors declare that the research was conducted in the absence of any commercial or financial relationships that could be construed as a potential conflict of interest.

## Publisher’s Note

All claims expressed in this article are solely those of the authors and do not necessarily represent those of their affiliated organizations, or those of the publisher, the editors and the reviewers. Any product that may be evaluated in this article, or claim that may be made by its manufacturer, is not guaranteed or endorsed by the publisher.
