# Supplementary material for: Barriers to Uptake of Open-Source Automated Insulin Delivery Systems: Analysis of Socioeconomic Factors and Perceived Challenges of Caregivers of Children and Adolescents With Type 1 Diabetes From the OPEN Survey
Source: Front Clin Diabetes Healthc. 2022 Jul 25;3:876511. doi: 10.3389/fcdhc.2022.876511 (PMC10012142; doi:10.3389/fcdhc.2022.876511)
Supplement: Supplementary file 1 [file DataSheet_1.docx]

**Supplementary Figure 1:** Distribution of participant groups of the OPEN survey. In total, 1052 individuals participated. They were divided into users (n=718) and non-users (n=257). Of them, 76 dropped out before completing any of the questionnaires. Participants were further divided into adults, adolescents, caregivers of children with diabetes, and partners of people with diabetes. Of the non-users, 56 were caregivers of children or adolescents with diabetes, which is the sub-population of this analysis.


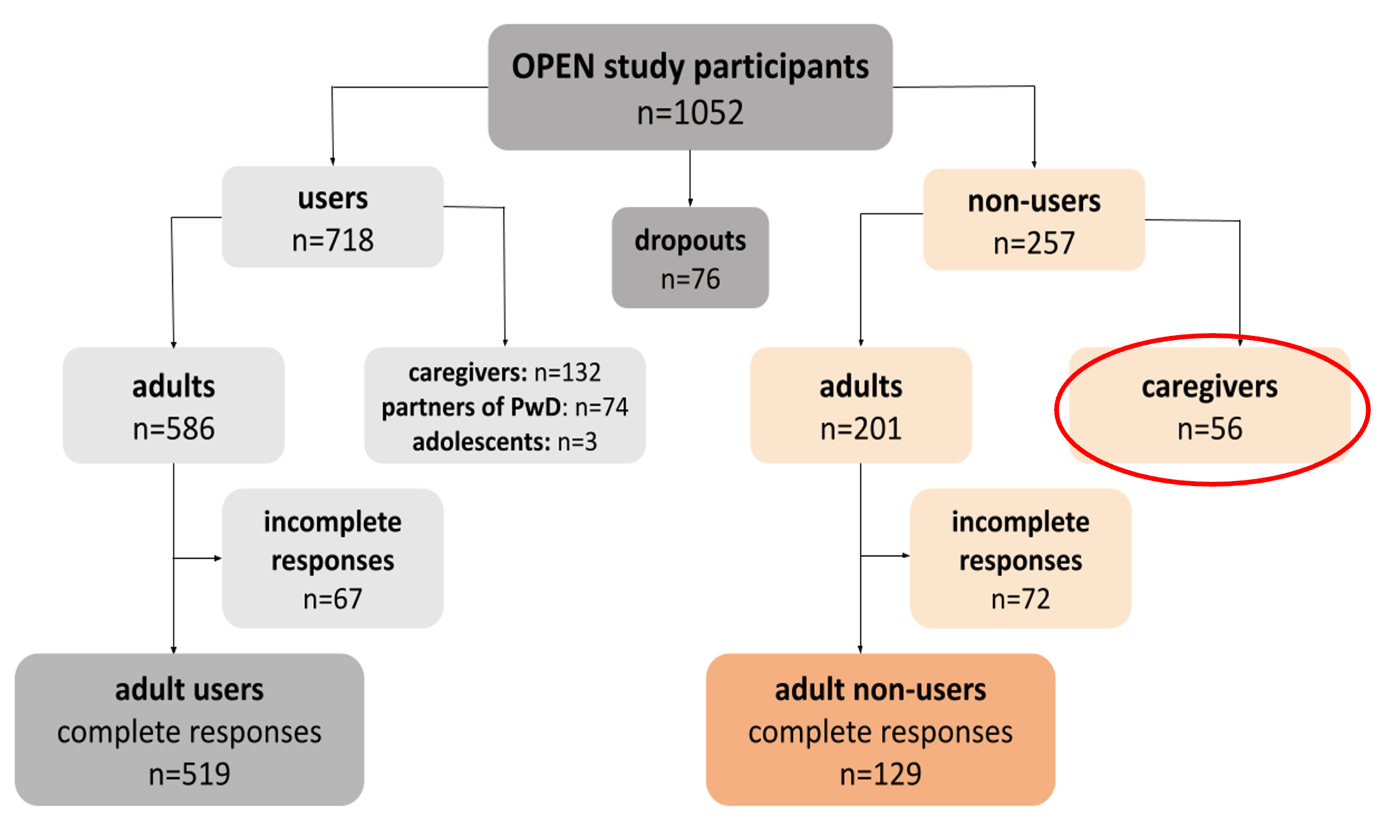


**Supplementary Table 1:** Demographic data of the participants and their child with diabetes (N=56).

| **Child's gender  [% (n)]** | Female | 20 (41) |
| --- | --- | --- |
|  | Male | 29 (59) |
|  | Other | 0 (0) |
|  | I'd rather not say | 0 (0) |
| **Child's birth county  [% (n)]** | **Europe** | **79,5 (39)** |
|  | Croatia | 6,1 (3) |
|  | Denmark | 22,4 (11) |
|  | Finland | 2 (1) |
|  | Germany | 24,5 (12) |
|  | Ireland | 8,2 (4) |
|  | Italy | 4,1 (2) |
|  | Netherlands | 2 (1) |
|  | United Kingdom | 10,2 (5) |
|  |  |  |
|  | **North America** | **12,2 (6)** |
|  | United States | 10,2 (5) |
|  | Bermuda | 2 (1) |
|  |  |  |
|  | **Australia and Oceania** | **6,1 (3)** |
|  | Australia | 2 (1) |
|  | New Zealand | 4,1 (2) |
|  |  |  |
|  | **Middle East** | **2 (1)** |
|  | Saudi Arabia | 2 (1) |
| **Child's ethnicity [% (n)]** | American Indian or Alaska Native | 0 (0) |
|  | Asian | 0 (0) |
|  | Arab | 2 (1) |
|  | Black or African American | 0 (0) |
|  | Hispanic/Latino | 2 (1) |
|  | Native Hawaiian or other Pacific Islander | 2 (1) |
|  | White | 87,8 (43) |
|  | Mixed/Multiple ethic groups | 0 (0) |
|  | Other | 4,1 (2) |
|  | I'd rather not say | 2 (1) |
| **Caregiver employment status [% (n)]** | Employed (full or part-time) | 63,3 (31) |
|  | Unemployed | 6,1 (3) |
|  | Retired | 0 (0) |
|  | Student/Apprentice | 6,1 (3) |
|  | None of the above | 18,4 (9) |
|  | I'd rather not say | 6,1 (3) |
| **Caregiver work sector [% (n)]** | Healthcare & Medicine | 16,3 (8) |
|  | Accountancy, Banking & Finance | 2 (1) |
|  | Business, Consulting & Management | 10,2 (5) |
|  | Engineering & Manufacturing | 10,2 (5) |
|  | Law | 0 (0) |
|  | Life Science | 0 (0) |
|  | Research | 2 (1) |
|  | Education | 4,1 (2) |
|  | Social Care | 8,2 (4) |
|  | Pharmaceutical or medical device industry | 6,1 (3) |
|  | Computer and Electronics | 0 (0) |
|  | Telecommunications | 8,2 (4) |
|  | Information Services and Data Processing | 0 (0) |
|  | Service | 10,2 (5) |
|  | Other | 8,2 (4) |
|  | I don't know | 12,2 (6) |
|  | I'd rather not say | 2 (1) |
| **Annual household income [% (n)]** | < 20,000 USD | 6,1 (3) |
|  | 20,000 to 34,999 USD | 2 (1) |
|  | 35,000 to 49,999 USD | 12,2 (6) |
|  | 50,000 to 99,999 USD | 20,4 (10) |
|  | 100,000 to 199,999 USD | 32,7 (16) |
|  | > 200,000 USD | 6,1 (3) |
|  | I don’t know | 0 (0) |
|  | I’d rather not say | 20,4 (10) |
| **Caregiver education: highest completed [% (n)]** | Less than a high school diploma | 2 (1) |
|  | High school degree or equivalent | 18,4 (9) |
|  | Some college, no degree | 2 (1) |
|  | Associate degree (e.g. AA, AS) | 2 (1) |
|  | Bachelor´s degree or equivalent level (e.g. BA, BS) | 30,6 (15) |
|  | Master´s degree or equivalent level (e.g. MA, MS, Med) | 34,7 (17) |
|  | Professional degree or equivalent level (e.g. MD, DDS, DVM) | 4,1 (2) |
|  | Doctorate (e.g. PhD, EdD) | 4,1 (2) |
|  | I'd rather not say | 2 (1) |
